# Microbiology and management of *Staphylococcus aureus* lacrimal system infections: A 10-year retrospective study

**DOI:** 10.1371/journal.pone.0314366

**Published:** 2024-11-22

**Authors:** Niloufar Bineshfar, Kevin D. Clauss, Wendy W. Lee, Darlene Miller

**Affiliations:** 1 Department of Ophthalmology, Bascom Palmer Eye Institute, University of Miami Miller School of Medicine, Miami, FL, United States of America; 2 Ocular Microbiology Laboratory, Bascom Palmer Eye Institute, Anne Bates Leach Eye Center, University of Miami Miller School of Medicine, McKnight Research Pavilions, Miami, FL, United States of America; University of Calgary, CANADA

## Abstract

**Purpose:**

To assess the *in vitro* efficacy of common antimicrobial agents used empirically for methicillin- resistant and sensitive *Staphylococcus aureus* (MRSA and MSSA) infections of the lacrimal system.

**Methods:**

A retrospective review of culture-proven *S*. *aureus* isolates retrieved from lacrimal system samples collected between January 2013–December 2022 was performed. Microbiologic characteristics such as *in vitro* susceptibility as well as clinical characteristics including history of recent ocular surgery, presence of lacrimal biomaterial implant, anti-microbial regimen, and treatments outcome were collected.

**Results:**

One hundred and sixteen *S*. *aureus* isolates (patients = 116) were identified. Thirty-one (27.4%) and 22 (19.5%) patients had recent ocular procedure and lacrimal intubation, respectively. Fifty (44.2%) patients received a combination of oral and topical antibiotics as first line of treatment. The most common empirically utilized antibiotics were β-lactams (38.9%) and polymyxin B/ trimethoprim (31.0%). The antibiotic regimen was changed at least once in 20.5% of patients due to ineffectiveness. Of the patients with positive cultures from the lacrimal excretory apparatus, 37.3% underwent surgery as part of the treatment approach. Of all isolates identified 44.8% were MRSA. Among the fluoroquinolones, the resistance rate was 38.8% for ciprofloxacin and 30.4% for moxifloxacin, with significantly higher resistance rates in MRSA (*P*-value <0.0001). The resistance rates for trimethoprim/sulfamethoxazole (TMP/SXT) and gentamicin were 8.6% and 3.4%, retrospectively.

**Conclusions:**

There is low *in vitro* efficacy of commonly used antimicrobials such as β-lactams and fluoroquinolones in our study population; thus, we recommend opting for trimethoprim/sulfamethoxazole and gentamicin for systemic and topical single-agent treatments.

## Introductions

The lacrimal apparatus is responsible for the production and drainage of tears and consists of the secretory and excretory systems. The excretory apparatus includes the anatomical structures responsible for tear clearance from the ocular surface and drainage into the nasal cavity. It consists of lacrimal punctum on the margin of the upper and lower eyelids, which open into the superior and inferior canaliculi, which typically join into a common canaliculus, which then drain into the lacrimal sac that is contiguous with the nasolacrimal duct within the canal of the maxillary bone, before draining into the inferior meatus of the nasal cavity [1].

The pathological colonization of bacteria in the lacrimal excretory apparatus can lead to dacryocystitis and canaliculitis, which manifest clinically as eye injection, discharge, epiphora, punctal inflammation (“pouting punctum”), as well as periorbital edema, erythema, tenderness and warmth. If left untreated or not treated promptly, the infection could lead to abscess formation, pre-septal cellulitis, and post-septal orbital cellulitis. Rarely, severe infections can spread to adjacent periorbital structures and cause life threatening conditions such as meningitis and cavernous sinus thrombosis [2–5].

Staphylococcal species are the most common virulent microorganisms that cause lacrimal apparatus infections, with *Staphylococcus aureus* (*S*. *aureus*) accounting for 23.8 to 30% of dacryocystitis cases [6–9]. A recent retrospective study, which included 195 bacterial isolates from the lacrimal drainage system, reported that *S*. *aureus* was identified in more than half of lacrimal isolates [10]. Previous studies on lacrimal system infections have reported various methicillin resistance rate, ranging from 20% to 43.9% [6,8,10]. Methicillin-resistant *Staphylococcus aureus* (MRSA) is known for causing high mortality worldwide due to its multi-drug resistance and the rapid progression of disease subsequent to infection. MRSA can be identified by detecting the methicillin resistance gene (*mecA*), which encodes a methicillin-resistant penicillin-binding protein [11].

Various antibiotics are utilized to treat *S*. *aureus* infections. However, in the era of increasing antibiotic resistant organisms, appropriate antibiotic selection can be challenging, as there is increased risk of treatment failure. Many antibiotics are prescribed both topically and systemically to treat a wide variety of other bacterial infections, often just prophylactically or inappropriately used, increasing the risk of cross-resistance and treatment failure. For instance, besifloxacin, a fluoroquinolone used for bacterial conjunctivitis treatment and formulated only for topical ophthalmic use, has lower resistance rates compared to other fluoroquinolones that are prescribed systemically. However, the reported *in vitro* resistance rates are still high [12,13].

Given the marked prevalence of multi-drug resistant subtypes and the empirical treatment of lacrimal apparatus infections, it is important to determine the changes in the prevalence of multi-drug resistant subtypes and resistance rates of the most common bacterial isolates, especially MRSA. In this retrospective study we aim to identify the prevalence of MRSA in lacrimal system isolates and *in vitro* anti-microbial resistance rates among MRSA versus MSSA samples at Bascom Palmer Eye Institute.

## Materials and methods

### Study design

A retrospective review of culture-proven *S*. *aureus* isolates recovered from lacrimal system samples collected between January 2013—December 2022 was performed. Institutional review board approval was obtained from the University of Miami, Miller School of Medicine Sciences Subcommittee for the Protection of Human Subjects (IRB Protocol Study ID #200709). The study was conducted in accordance with the tenets of the Declaration of Helsinki. It was compliant with the provisions of the United States of America Health Insurance Portability and Accountability Act of 1996 (HIPAA). The isolates were identified by searching the Ocular Microbiology Department’s database (Table 1). In patients with persistent infection and more than one *S*. *aureus* positive culture (n = 8) only the characteristics of the first isolate were recorded. However, the resistance patterns were similar across all cultures, except for one patient who had both MSSA and MRSA.

**Table 1:** Specimens’ details.

Specimen source	All isolates, n	*S*. *aureus* isolates, n	*S*. *aureus* prevalence
**Lacrimal sac and nasolacrimal duct**	289	96	33.2%
**Canaliculi**	79	20	25.3%
**All sites**	368	116	31.5%

### Data collection

Microbiologic data such as isolate, specimen source, and *in vitro* susceptibility were recorded. Patients’ medical records were reviewed to extract clinical data including gender, age, clinical setting, history of diabetes, history of recent ocular surgery, presence of lacrimal biomaterial implant, symptom onset, and treatment details.

### Antibiotic susceptibility test

Primary clinical specimens from infection site were collected by the attending ophthalmologist or resident according to institutional guidelines and submitted to the clinical microbiology laboratory for processing. Samples were inoculated following standard microbiological procedures. Each sample was inoculated onto 5% sheep blood agar plate, chocolate agar plate, and thioglycolate broth, then incubated aerobically at 35°C for up to 7 days. Fungi were cultured on Sabouraud agar, both with and without chloramphenicol, at 25°C for 14 days. Gram staining was performed routinely.

S. aureus identification was confirmed with the BactiStaph Latex kit (Remel, Lenexa, KS, US). Isolates were classified as MRSA vs MSSA using the cefoxitin screen and oxacillin susceptibility. These along with *in vitro* resistance patterns (MIC ug/ml) were determined using the automated system Vitek 2 (BioMériuex, Durham, NC). Although susceptibility breakpoints are not established for the topical treatment of ocular infections with eye drops, given that the majority of lacrimal system infections were treated systemically, results were interpreted according to Clinical Laboratory Standard Institute (CLSI) guidelines [14]. The isolates were classified as S (susceptible), I (intermediate), and or R (resistant). Advanced Expert System (AES) of the VITEK 2 was used to infer a genotype (resistance mechanism) from the susceptibility patterns.

### Microtiter plate assay

The biofilm formation was assessed in a subgroup of isolates (n = 29). The degree of biofilm production was determined by a microtiter plate assay as previously described [15]. Bacteria were inoculated into tryptic soy broth (TSB) with 0.25% glucose (Remel, Lenexa, KS, US) and incubated overnight at 37°C. For each isolate a 0.5 McFarland suspension, approximately 1 x 10^8^ colony-forming units (CFU)/ml was prepared, and 150 μl of solution was dispensed into each well of 96-well polystyrene microtiter plate (Costar 3599, Corning, Tissue Culture- Treated; Corning Inc., Corning, NY). After 24 hours incubation at 37°C under aerobic conditions, the plates were washed three times with distilled water and stained with 0.1% crystal violet, for 15 minutes. The staining was washed with distilled water. After solubilizing the stain by adding 180 μl of 30% acetic acid in water to each well, the quantity of adherent biofilm was determined by measuring the optical density (OD) at 550 nm. A well with 30% acetic acid in water served as blank. We used triplicates for each isolate and the isolate OD calculated by subtracting the negative control OD from the average OD of the three replicates. The interpretation and categorization of the results have been described previously [16]. The cut-off value (ODc = average OD of negative control+(3×standard deviation (SD) of negative control)) was calculated and the OD of each isolate was categorized based on ODc. (OD ≤ODc = no biofilm producer; ODc <OD ≤2×ODc = weak biofilm producer; 2×ODc <OD ≤4×ODc = moderate biofilm producer; 4×ODc <OD = strong biofilm producer).

### Statistical analysis

The antimicrobials susceptibility was compared between methicillin-resistant and susceptible isolates and the first five-year (n = 64) and the second five-year (n = 52) study period. All statistical analyses were performed using SPSS (version 26.0; IBM, Armonk, NY, USA) and GraphPad Prism (version 9.5.1; GraphPad, La Jolla, CA, USA). Continuous variables were shown as mean ± standard deviation (SD). Categorical variables were presented as count (percentage). The normality of continuous variables was tested by the Kolmogorov-Smirnov test. Logistic regression used to calculate the odds ratios (ODs) for resistance to each antibiotic. Student t-test and Mann-Whitney U test were applied to analyze parametric and non-parametric continuous variables, respectively. Categorical variables were analyzed using Chi-square or Fisher’s exact test. Significance was set at *P*-value of < 0.05. and ns, *, **, ***, and **** represent *P*-value >0.05, <0.05, <0.01, <0.001, and <0.0001, respectively.

## Results

### Patients characteristics

During the 10-year duration of the study, 368 specimens were collected from lacrimal apparatus and 116 *S*. *aureus* isolates were identified among them. Patients’ characteristics are summarized in Table 2. The mean age of the patients was 57.5±24.8 (range, 1–108) years, and 72 (62.1%) were females. The prevalence of recent antibiotic use was 39.5%. Thirty-one (27.4%) and 22 (19.5%) patients had recent ocular surgery and stent lacrimal intubation, respectively. All the lacrimal biomaterials (100%) were removed as part of the treatment. Of the patients with cultures from the lacrimal excretory apparatus, 41 (37.3%) patients underwent surgery, including 33 dacryocystorhinostomy (DCR) and 8 canaliculi surgeries. The canaliculi surgery included canaliculoplasty (n = 2), marsupialization of canaliculi (n = 2), punctoplasty (n = 1), canaliculostomy (n = 1), and canaliculotomy (n = 1).

**Table 2:** Patients’ characteristics.

Characteristics	Total, n = 116	MSSA, n = 64	MRSA, n = 52	*P*-value
**Age, mean ± SD, years**	57.5±24.8	55.3±25.6	60.2±24.1	0.300
**Female Sex, No. (%)**	72 (62.1)	37 (57.8)	35 (67.3)	0.295
**Clinical presentation, No. (%)**				0.291
Acute	62 (55.4)	31 (50.8)	31 (60.8)	
Non-acute	50 (44.6)	30 (49.2)	20 (39.2)	
**Symptom onset** ^a^ **, mean ± SD, days**	4.9±4.9	5.7±6.3	4.0±2.6	0.957
**Specimen source, No. (%)**				0.315
Lacrimal sac and nasolacrimal duct	96 (82.8)	55 (85.9)	41 (78.8)	
Canaliculi	20 (17.2)	9 (14.1)	11 (21.2)	
**Past Medical History, No. (%)**				–
Diabetes mellitus	20 (17.5)	11 (17.2)	9 (18.0)	0.910
Malignancy	13 (11.4)	10 (15.6)	3 (6.0)	0.109
Previous NLDO	20 (17.5)	14 (21.9)	6 (12.0)	0.169
Recent ocular surgery	31 (27.4)	26 (41.3)	5 (10.0)	**<0.001**
**Lacrimal biomaterial**	22 (19.5)	17 (30.4)	5 (11.1)	0.305
Bicanalicular stent (Silicone)	13 (11.2)	11 (17.7)	2 (3.9)	–
Jones tube (Pyrex glass)	2 (1.7)	2 (3.2)	0	–
Punctal plugs	7 (6.0)	4 (6.5)	3 (5.9)	–
**Recent antibiotic use, No. (%)**	45 (39.5)	27 (43.5)	18 (34.6)	0.331
**First line treatment details, No. (%)**				0.106
Topical antibiotics	28 (24.8)	16 (25.0)	12 (24.5)	
Oral antibiotics	35 (31.0)	15 (23.5)	20 (40.8)	
A combination of topical and oral	50 (44.2)	33 (51.6)	17 (34.7)	
**Persistent infection** ^b^ **, No. (%)**	23 (20.5)	12 (18.8)	11 (22.9)	0.589
**Surgical treatment, No. (%)**	41	26	15	0.250
DCR	33 (30.0)	22 (35.5)	11 (22.9)	–
Canaliculi surgery	8 (7.3)	4 (6.5)	4 (8.3)	–

NLDO, nasolacrimal duct obstruction; DCR, dacryocystorhinostomy.

^a^Calculated for acute presentations.

^b^Patients that were unresponsive to the first antibiotic treatment and required a change in antibiotic regimen.

### Antimicrobials

**Fig 1:**
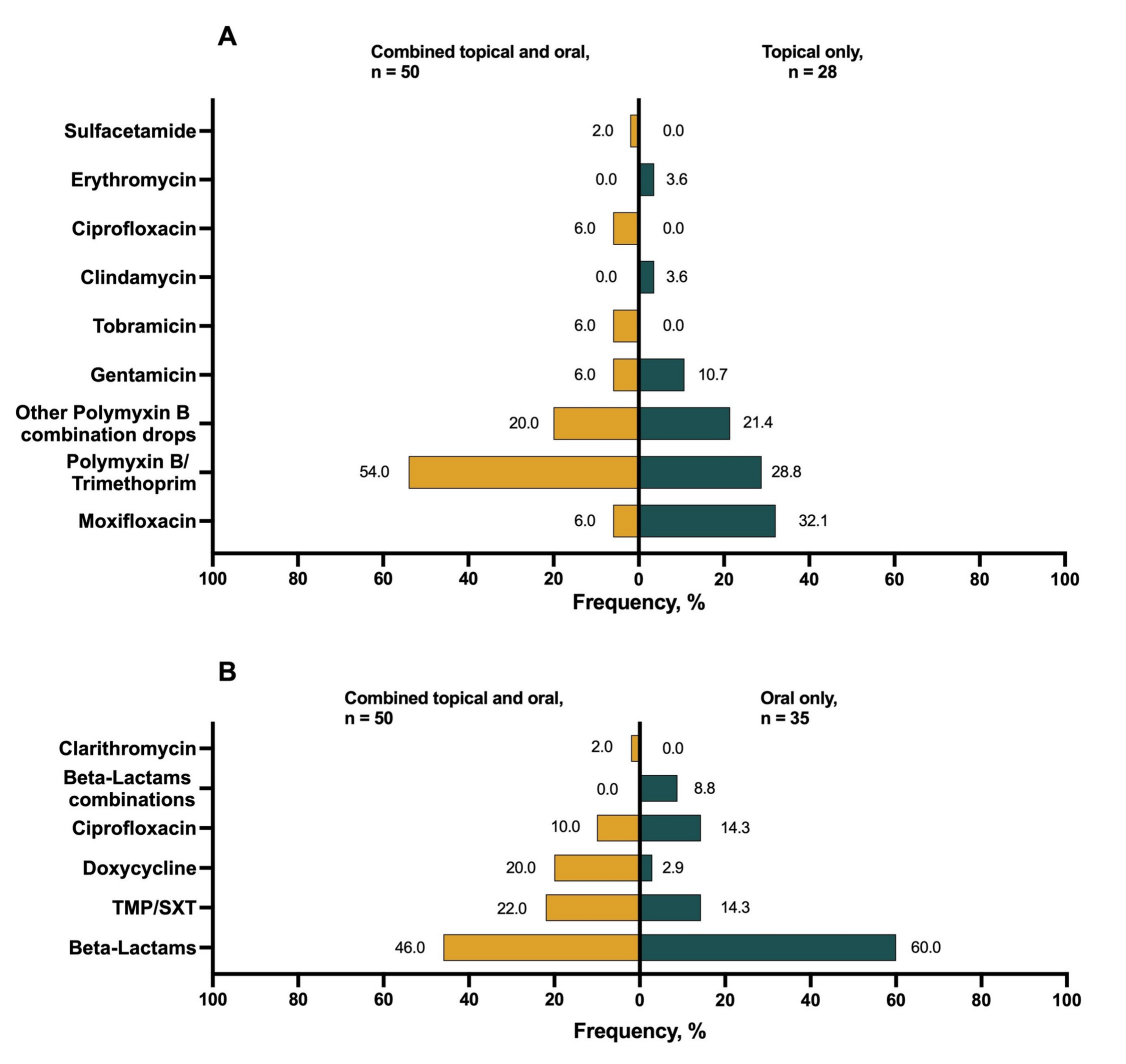
The prevalence of antibiotics used as the first line of treatment. A: The percentage of topical antibiotics used as a combined or an only topical regimen. B: The percentage of oral antibiotics used as part of a combined or an only oral regimen.

The most common first line of treatment was a combination of oral and topical antibiotics (n = 50, 44.2%). The remainder of patients received the antimicrobials from only one route either oral (n = 35, 31.0%) or topical antibiotics (n = 28, 24.8%). The most common antibiotics utilized were β-lactams (n = 44, 38.9%) and polymyxin B/ trimethoprim (n = 35, 31.0%). Among patients who were prescribed oral antibiotics only, 21 (60.0%) were treated with a β-lactam. Moreover, among patients treated with topical antibiotics, 9 (32.1%) received moxifloxacin. The treatment details are shown in Fig 1.

### Resistance

Of the total 116 isolates identified 52 (44.8%) were methicillin-resistant. Among the fluoroquinolones, the resistance rate was 38.8% for ciprofloxacin, 38.8% for levofloxacin, and 30.4% for moxifloxacin. The resistance rates for trimethoprim/sulfamethoxazole (TMP/SXT), tetracycline, gentamicin, and vancomycin were 8.6%, 12.1%, 3.4%, and 0.9%, retrospectively. All the isolates were susceptible to doxycycline. Antibiotic resistance and MICs are shown in Table 3.

**Table 3:** Antibiotics sensitivity and minimum inhibitory concentration details.

Antibiotics	Total, n = 116				MSSA, n = 64				MRSA, n = 52				Concurrent drug resistance with MRSA	
	MIC, μg/ml			Resistance, %	MIC, μg/ml			Resistance, %	MIC, μg/ml			Resistance, %		
	Range	MIC_50_	MIC_90_		Range	MIC_50_	MIC_90_		Range	MIC_50_	MIC_90_		OR	95% CI
**Erythromycin**	0.25–8.0	8.0	8.0	62.9	0.25–8.0	0.25	8.0	40.6	0.25–8.0	8.0	8.0	90.4	**13.74**	4.8–39.19
**Clindamycin**	0.12–4.0	0.25	4.0	29.3	0.12–4.0	0.25	0.38	29.7	0.25–4.0	0.25	4.0	28.8	0.96	0.43–2.15
**Ciprofloxacin**	0.5–8.0	0.5	8.0	38.8	0.5–8.0	0.5	8.0	17.2	0.5–8.0	8.0	8.0	65.4	**9.10**	3.83–21.61
**Levofloxacin**	0.12–8.0	0.25	8.0	38.8	0.12–8.0	0.25	6.0	17.2	0.12–8.0	4.0	8.0	65.4	**9.10**	3.83–21.61
**Moxifloxacin**	0.25–8.0	0.25	8.0	30.4	0.25–8.0	0.25	2.0	11.1	0.25–8.0	2.0	8.0	53.8	**9.33**	3.59–24.29
**Oxacillin**	0.25–4.0	0.5	4.0	44.8	0.25–1.0	0.5	0.5	0.0	0.5–4.0	4.0	4.0	100	–	–
**TMP/SXT**	10.0–320.0	10.0	10.0	8.6	10.0–320.0	10.0	10.0	4.7	10.0–320.0	10.0	320.0	13.5	3.16	0.78–12.91
**Tetracycline**	1.0–16.0	1.0	16.0	12.1	1.0–16.0	1.0	16.0	12.5	1.0–16.0	1.0	16.0	11.5	0.91	0.30–2.82
**Doxycycline**	0.5–4.0	0.5	1.5	0.0	0.5–4.0	0.5	1.3	0.0	0.5–4.0	0.5	2.0	0.0	–	–
**Gentamycin**	0.5–16.0	0.5	0.5	3.4	0.5–16.0	0.5	0.5	1.6	0.5–16.0	0.5	0.5	5.8	3.86	0.39–38.23
**Vancomycin**	0.5–32.0	1.0	1.0	0.9	0.5–2.0	0.1	0.1	0.0	0.5–32.0	1.0	1.0	1.9	–	–

MSSA, Methicillin-sensitive *Staphylococcus aureus; MRSA*, Methicillin-resistant *Staphylococcus aureus;* MIC, minimum inhibitory concentration; OR, odds ratio; CI, confidence interval.

#### Resistance in MRSA versus MSSA

**Fig 2:**
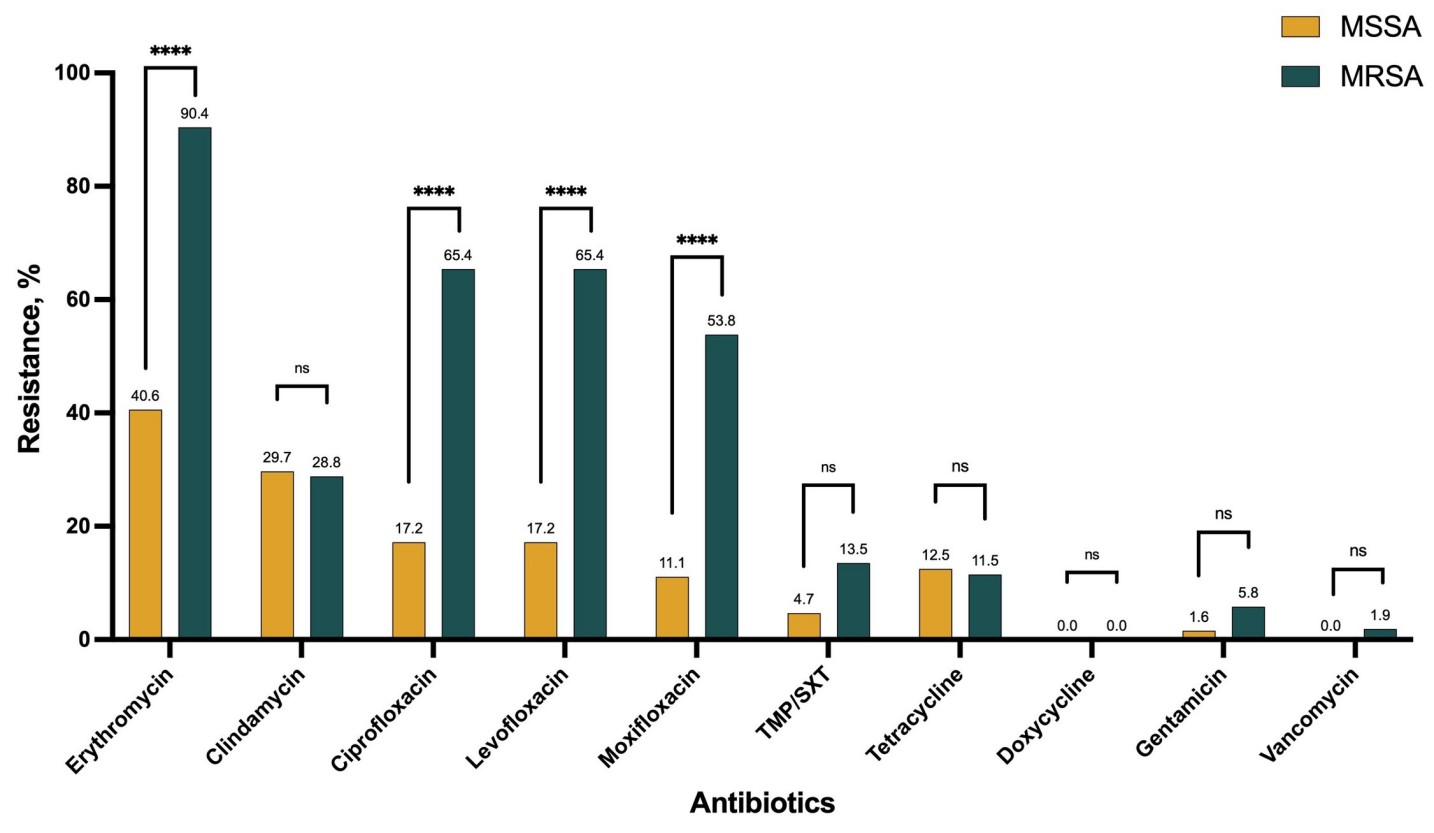
Antimicrobials resistance in MSSA vs MRSA. MRSA, Methicillin-sensitive Staphylococcus aureus; MRSA, Methicillin-resistant Staphylococcus aureus.

The resistance rates for fluoroquinolones were significantly higher among methicillin-resistant *Staphylococcus aureus* (MRSA) compared to methicillin-sensitive *Staphylococcus aureus* (MSSA) (*P*-value<0.0001); for ciprofloxacin 65.4% and 17.2%; for levofloxacin 65.4% and 17.2%; and for moxifloxacin 53.8% and 11.1%, respectively. In addition, compared to MSSA, MRSA had higher resistance against erythromycin (*P*-value<0.0001). For the other antimicrobials, the susceptibility did not differ between MRSA and MSSA isolates (Fig 2).

#### Vancomycin MIC

The MIC for vancomycin was ≥1 μg/ml in 95 (81.9%) of patients, among them four had an MIC of 2 μg/ml and one had an MIC of 32 μg/ml. The prevalence of MIC of ≥1 μg/ml was 50 (78.1%) and 45 (86.5%) patients among MSSA and MRSA isolates, respectively.

#### Resistance changes over time

**Fig 3:**
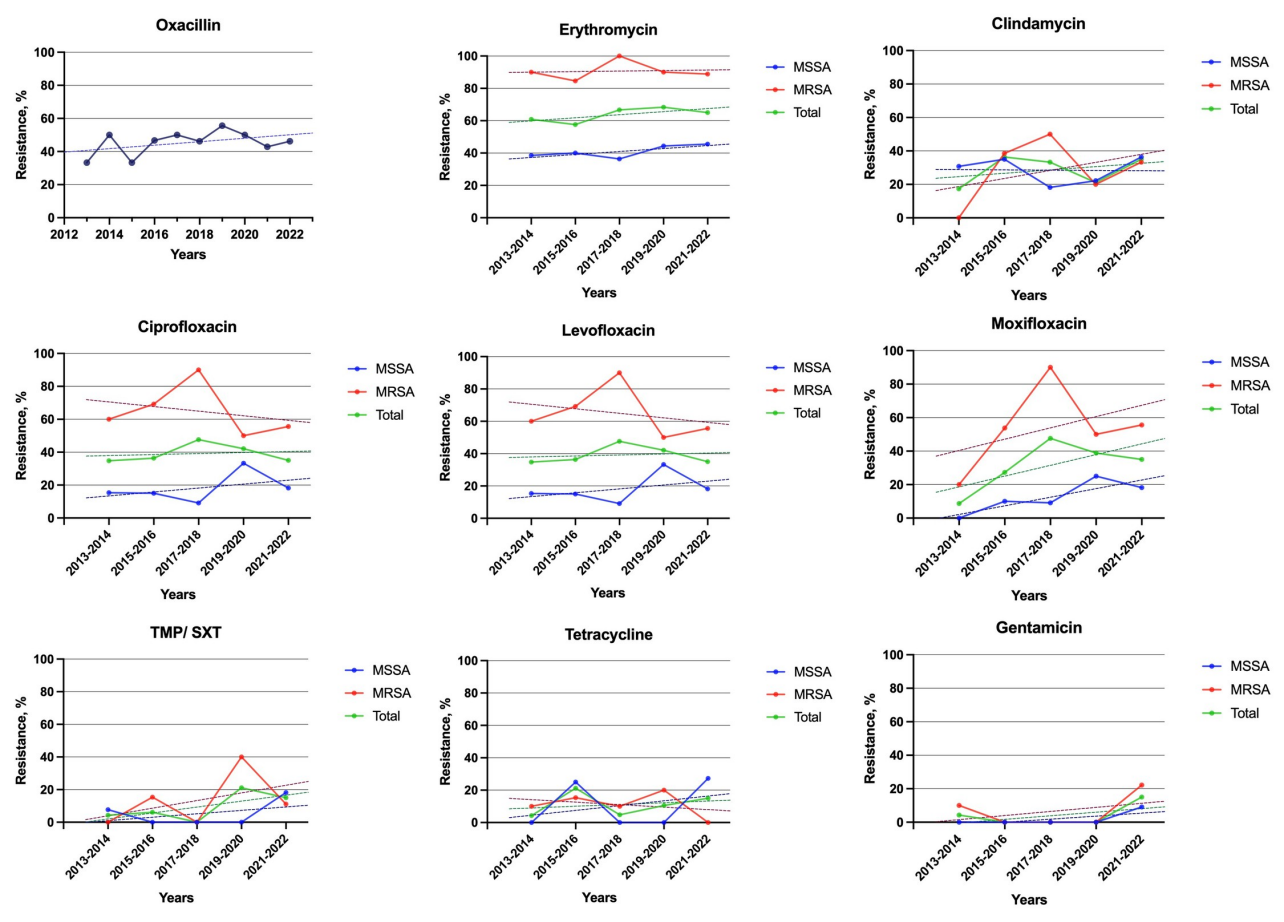
Resistance trends during the study period among MSSA and MRSA isolates. All the slopes were not significant (P-value>0.5). MRSA, Methicillin-sensitive Staphylococcus aureus; MRSA, Methicillin-resistant Staphylococcus aureus; TMP/SXT, trimethoprim sulfamethoxazole.

**Fig 4:**
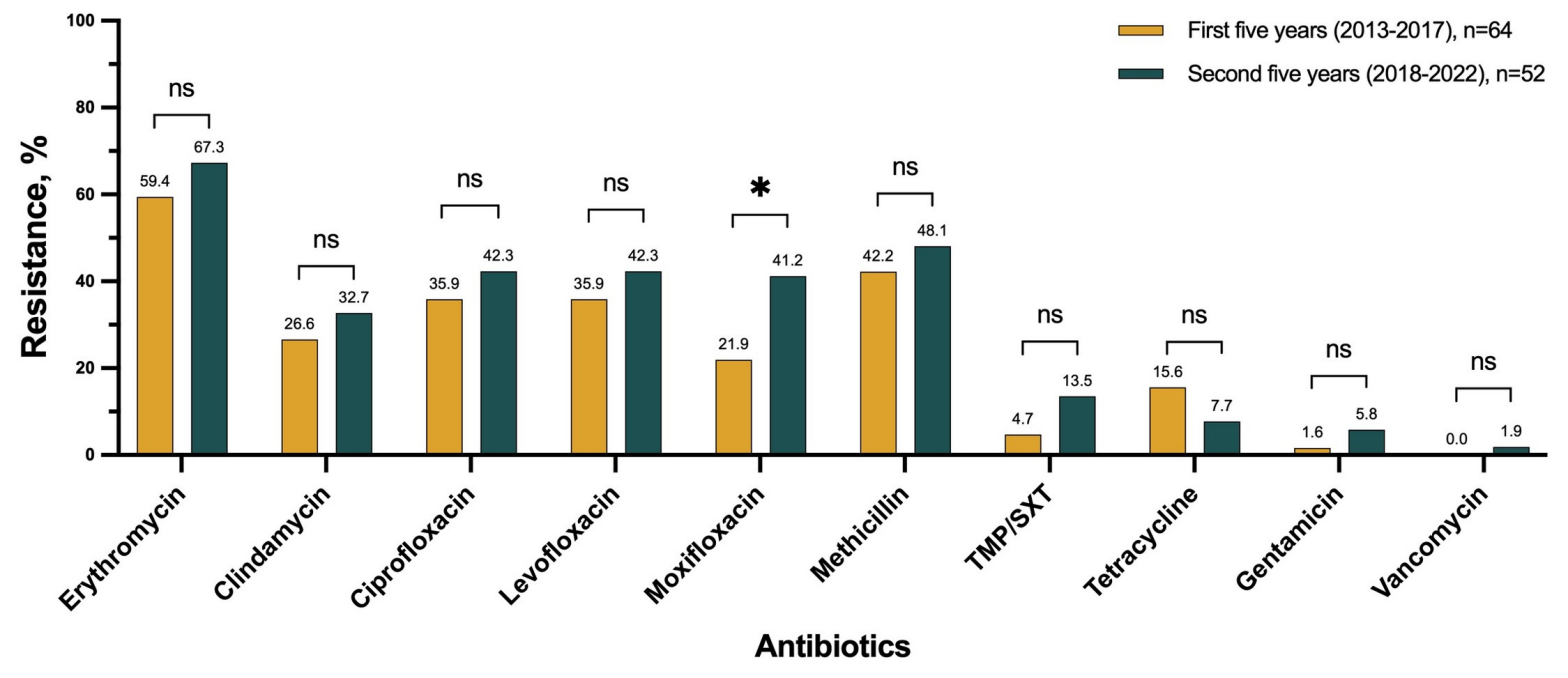
Resistance to antibiotics in different study periods.

Overall, for most antibiotics, resistance showed a stable trend during the study period (*P*-value>0.05) (Fig 3). When comparing the resistance rates between the first (2013–2017) and the second half of the study period (2018–2022), the highest resistance change was observed for moxifloxacin, with an increase from 21.9% to 41.2% (*P*-value = 0.025). The resistance change for other antibiotics was non-significant (Fig 4).

#### Mechanism of resistance

*mecA* is the gene that confers methicillin resistance. In our survey, genotypic methicillin resistance correlated with the phenotypic resistance. It should be noted that these genotype or resistance mechanisms were identified based on the interpretation of MIC values. However, it is widely accepted that using VITEK 2 AES allows for reliable detection of resistance related to β-lactams [17]. Additionally, multicenter evaluations have shown that VITEK 2 AES provides reliable phenotypic interpretations for quinolones and aminoglycosides [18]. Furthermore, all isolates with gentamicin resistance had *aac(6’)-aph(2”)* gene (Table 4).

**Table 4:** Genotype or resistance mechanisms detected by VITEK 2.

Antibiotic	Genotypic resistance			
	Genotype	Total, n = 116	MSSA, n = 64	MRSA, n = 52
**Quinolones**	***Resistant***	12	3	9
	***Partially resistant***	34	9	25
**β-lactam**	***mecA***	51	–	51
	***Acquired penicillinase***	65	64	1
**Gentamicin**	***aac(6’)-aph(2”)***	4	1	3

### Biofilm producing

Biofilm productions of isolates are categorized in Table 5. The mean OD_550_ was 0.1873 ± 0.2013. The mean ODs of MSSA and MRSA were 0.1808 ± 0.1964 and 0.1868 ± 0.2155, respectively. This difference was not statistically significant (*P*-value = 0.783).

**Table 5:** Biofilm production categorization.

Degree of biofilm production	Total, n = 29	MSSA, n = 19	MRSA, n = 10
**No Biofilm**	22 (75.9%)	14	8
**Weak biofilm**	3 (10.3%)	2	1
**Medium biofilm**	2 (6.9%)	2	0
**Strong biofilm**	2 (6.9%)	1	1

## Discussion

*S*. *aureus* is the most common cause of dacryocystitis and a common cause of canaliculitis, which commonly leads to mucopurulent discharge, ocular surface irritation, and eyelid infectious signs such as erythema, edema, tenderness, and warmth. More serious infections can lead to abscess formation, pre-septal or post-septal cellulitis. Typically, lacrimal apparatus infections respond to systemic antibiotics, however some cases require canaliculotomy or abscess incision and drainage. Recurrence is common if the underlying etiology, typically nasolacrimal duct obstruction, is not addressed.

Traditionally, MRSA colonization and infections were believed to be related to risk factors such as indwelling catheters, immunocompromised status, diabetes, drug injection, living in long-term care facilities, or recent hospitalization. However, cases of community-acquired MRSA are increasing. Previous studies have documented the same trend in ocular microbiology [10]. Moreover, our study shows compatible results with this theory, demonstrating that MRSA is not associated with typical risk factors as was anticipated. Many patients with MRSA did not have classical risk factors for MRSA infections, for instance only 11.1% had lacrimal biomaterials. In contrast patients with MSSA had significantly higher rates of prior history of ocular surgery and lacrimal intubation.

Although not significant, 60.8% of infections caused by MRSA were acute. This result is comparable with previous studies in which MRSA was primarily isolated from acute infections [6].

Biofilms are known to be antibiotic resistant. In our study, all the lacrimal biomaterials were removed as part of the treatment, highlighting the role of biofilm formation in the lacrimal system infections outcome. Previous studies have confirmed the presence of biofilm in the majority (83%) of infected periocular and orbital implants with electron microscopy [19,20]. In our study the degree of biofilm production did not differ between MSSA and MRSA isolates and only a small percent (13.8%) of isolates were moderate to strong biofilm producers.

Among patients with dacryocystitis, about one-third required DCR surgery. This emphasizes the importance of resolution of NLDO, which could result in infection reoccurrence if not addressed [6]. In the case of canaliculitis, restoration of patency is important, often times with canaliculotomy to remove infectious dacryoliths. Moreover, it has been reported that recanalization and restoring tear flow would decrease the virulent bacteria conjunctival sac and normalize conjunctival flora [21].

The methicillin/oxacillin resistance rate in our population (44.2%) was higher than most previously reported in the USA [10,22]; a reason for this is the geographic differences. As reported in ARMOR studies, southern states of the USA have a higher resistance rate for methicillin/oxacillin than the country’s average (~40% versus 34.9%) [23,24]. Another reason is the higher methicillin/oxacillin resistance rates among lacrimal system infections compared to other ocular infections [10].

In our study, there was no difference regarding resistance of clindamycin, trimethoprim/sulfamethoxazole (TMP/SXT), gentamicin, and vancomycin between MRSA and MSSA. However, previous studies have reported a high *in vitro* resistance rate in *S*. *aureus* of ocular isolates to fluoroquinolones, macrolides, and methicillin, with a higher rate of multi-drug resistance among MRSA [25].

Based on the published literature, erythromycin has co-resistance with methicillin. Andre et al. have reported a strong association between methicillin and erythromycin resistance, with a resistance rate of 88.4% and 40.2% with MRSA and MSSA, respectively [10]. Similarly, in our study, the resistance rate for macrolides was significantly higher among MRSA compared to MSSA isolates (89.1% versus 39.7%).

In the ARMOR study, the resistance rate for fluoroquinolones was 7.1–10.8% and 58.3–72.7% among MSSA and MRSA isolates, respectively [23]. Also, as reported in a systematic review, the susceptibility rates for the fluoroquinolones were lower among MRSA than MSSA (4.3–55.0% versus 79.9–96.0%) [25]. We observed similar results with lacrimal isolates. In our study the fluroquinolones resistance rates were significantly higher among MRSA.

Hetero-vancomycin intermediate *S*. *aureus* (hVISA) has a multistep generation course, evolving from vancomycin sensitive strains. The first mutation increases the vancomycin MIC to 2 μg/ml and following mutations cause progress to higher MICs [26]. In our population we observed four (3.4%) isolates with MIC of 2 μg/ml and one isolate that was resistant to vancomycin. Microbiology surveys have reported an increase in hVISA and VISA in recent years [27].

Generally, there is an increasing trend in resistance to β-lactams. In a study on keratitis isolates from 1993 to 2012, oxacillin resistance increased from the 18.4% in the first quarter to 38.3% in the last quarter of the study [28]. In the ARMOR study, oxacillin resistance among *S*. *aureus* isolates decreased by 2.16% per year between 2009 (39.0%) and 2018 (29.3%) [23]. In an eight-year survey on ocular isolates, the documented resistance rate was stable with an average of 28%. In our study the oxacillin resistance increased from 33.3% in the first two years (2013–2014) to 50.0% in the last two years (2021–2022).

Although the reported resistance rate for macrolides had an increasing trend with a rate of 3.74% (1997–2008) [29], the more recent trend was a 1.4% (2009–2018) annual reduction rate [23]. A more recent study reported a stable erythromycin resistance rate over the eight-year period of the study. At the beginning of the study, the resistance rates for MSSA and MRSA were 39.5% and 95.1%, respectively. By the end of the study, the rates had only slightly changed to 40.2% and 89.3% for MSSA and MRSA [10]. Similarly, we observed a stable resistance rate across the ten-year period of our study (R^2^ = 0.64, *P*-value = 0.103).

Most studies have reported increasing resistance rates over time for fluoroquinolones. A study at Bascom Palmer Eye Institute on *S*. *aureus* isolates from keratitis, and conjunctivitis reported an increasing trend in resistance to fluoroquinolones from 7.5 to 39.6% between 1990 and 2001 [30]. Similarly, another not recent study reported a 30% rise with a 2.57% annual increasing rate in fluoroquinolones resistance from 1997 to 2008 [29]. However, in the more recent ARMOR study fluoroquinolone resistance among *S*. *aureus* isolates had an annual decrease rate of 2.24% and they documented a drop in resistance rate between 2009 (38.5%) and 2018 (30.0%) [23]. In our study, the resistance rate for fluoroquinolones had an increasing trend including a dramatic increase in resistance to moxifloxacin from 9.1% in the first two years to 35.3% in the last two years.

A study on conjunctival isolates showed rising trends in resistance for some effective antimicrobials including TMP/SXT, tetracycline, and gentamicin [29]. However, our study findings indicate that the resistance trends for these antimicrobials have reached a plateau. Our findings align with recent reports [23].

In our study approximately one-fifth of the patients do not respond to the initial antibiotic therapy and require changing the antibiotic class. Another study has reported that about one-third of bacterial isolates are resistant to the initial antibiotic therapy [6].

In summary our data demonstrates the absence of traditional risk factors for developing MRSA infection. We identified treatment trends and a high level of resistance among *S*. *aureus* to some frequently used antibiotics in our clinic. Additionally, we presented resistance rates for commonly used antibiotics, revealing an increase in resistance for some, like fluoroquinolones, while others have reached a plateau in resistance change. Our data on methicillin/oxacillin resistance differs from previously published data, such as ARMOR; the reason could be the fact that the ARMOR data were not classified by infection type to allow evaluation of any potential disease/tissue-specific trends in the population analyzed. However, this discrepancy can be attributed to the differences in characteristics of isolates from ocular site and geographic locations.

The current study had some limitations, such as its retrospective nature, small sample size, and being single-centered. Additionally, it was not possible to retrieve all the samples and evaluate the biofilm formation ability of them. The strengths of this study include evaluating the clinical characteristics and treatments success rate along with *in vitro* susceptibilities to common antimicrobials. The study also documents a high rate of MRSA and evolving antibiotic resistance to fluoroquinolones among this study dataset.

## Conclusions

In conclusion, given the lack of evident risk factors for MRSA infection in some patients, ophthalmologists should always consider MRSA as an etiology for lacrimal apparatus infections, even in the absence of any risk factors. Considering the low *in-vitro* efficacy of frequently used antimicrobials such as β-lactams and fluoroquinolones in our study population, single-agent therapy with these antibiotics should be avoided. We recommend using TMP/SXT and doxycycline for systemic treatment, along with gentamicin for topical application.

## Supporting information

S1 File Study dataset. (XLSX)
